# The role of miR-10a-5p in LPS-induced inhibition of progesterone synthesis in goose granulosa cells by down-regulating CYP11A1

**DOI:** 10.3389/fvets.2024.1398728

**Published:** 2024-05-30

**Authors:** Xinyi Guo, Hao An, Rihong Guo, Zichun Dai, Shijia Ying, Wenda Wu

**Affiliations:** ^1^Ministry of Education Joint International Research Laboratory of Animal Health and Food Safety, College of Veterinary Medicine, Nanjing Agricultural University, Nanjing, China; ^2^Key Laboratory for Crop and Animal Integrated Farming, Ministry of Agriculture and Rural Affairs, Institute of Animal Science, Jiangsu Academy of Agricultural Sciences, Nanjing, China; ^3^School of Food and Biological Engineering, Hefei University of Technology, Hefei, China

**Keywords:** LPS, goose, miR-10a-5p, granulosa cells, progesterone

## Abstract

The poultry ovary is a preferred target for *E. coli* and *Salmonella* infection of tissues, and lipopolysaccharide (LPS) is a critical molecule in infecting the organism and interfering with cell function, invading the ovaries through the cloaca and interfering with progesterone (P4) secretion by follicular granulosa cells (GCs), seriously affecting the health of breeding geese. miRNAs are small, non-coding RNAs with a variety of important regulatory roles. To investigate the mechanism of miR-10a-5p mediated LPS inhibition of progesterone synthesis in goose granulosa cells, Yangzhou geese at peak laying period were selected as experimental animals to verify the expression levels of genes and transcription factors related to progesterone synthesis. In this study, bioinformatic predictions identified miR-10a-5p target gene CYP11A1, and genes and transcription factors related to the sex steroid hormone secretion pathway were screened. We detected that LPS inhibited CYP11A1 expression while increasing miR-10a-5p expression *in vivo*. Progesterone decreased significantly in goose granulosa cells treatment with 1 μg/mL LPS for 24 h, while progesterone-related genes and regulatory factors were also suppressed. We also determined that the downregulation of miR-10a-5p led to CYP11A1 expression. Overexpression of miR-10a-5p suppressed LPS-induced CYP11A1 expression, resulting in decreased progesterone secretion. Our findings indicated that miR-10a-5p was up-regulated by LPS and inhibited progesterone synthesis by down-regulating CYP11A1. This study provides insight into the molecular mechanisms regulating geese reproduction and ovulation.

## 1 Introduction

Lipopolysaccharide (LPS) is the main virulence factor of bacterial endotoxins released by the cell walls lysis of Gram-negative bacteria such as *E. coli and Salmonella*. The LPS molecule can be divided into O-antigen, core oligosaccharide, and Lipid A (1). LPS can significantly affect mammalian and avian physiological activities, reduce immune function, and inhibit growth performance (2). The effects of LPS on the reproductive system have been extensively studied in mammals. In mammals, LPS mediates ovarian inflammatory responses, reduces steroid production, increases apoptosis, and inhibits follicular and corpus luteum growth (3). Elevated LPS concentrations in dairy cows' follicular fluid led to increased expression of LPS receptor genes, which were significantly correlated with the expression of genes related to steroid hormone secretion in both follicular granulosa cells (GCs) and Theca cells (4). Additionally, LPS may affect STAR expression in porcine follicular granulosa cells through GATA4/6 transcription factors (5). Intensive goose farming practices are considered to be associated with disease and environmental pollution problems, with feces discharged into the water, leading to elevated concentrations of LPS in both the water and goose plasma (6). In addition, ovaries are susceptible to pathogenic bacteria due to specific mating behavior in pathogen-laden water, which may reduce egg production performance in geese (7). However, research on LPS in avian species has primarily focused on immunity and inflammation, with few studies investigating the mechanism of granulocyte progesterone synthesis.

In avian species, follicular maturation is an ongoing reproductive process that includes replenishment, growth, selection, and innervation (8, 9). At sexual maturity, follicles form a hierarchical system within the ovary based on their functional status, which means follicular development is a priority (10). In general, ovarian follicles can be divided into two categories: pre-hierarchical follicles and hierarchical follicles. Different stages of the follicle secrete various steroid hormones. Progesterone (P4) is a steroid hormone secreted exclusively by avian follicular granulosa cells and plays a key role in follicle development and ovulation. As follicles develop, E2 secretion gradually decreases while progesterone secretion gradually increases (11). The main site of progesterone synthesis in avian ovaries is the granulosa layer of the three largest yellow pre-ovulatory follicles (12) during the transition from pre-hierarchical to hierarchical follicles, luteinizing hormone (LH)-stimulated granulosa layer to express the cholesterol side-chain cleaving enzyme (CYP11A1) and begin to produce progesterone (13). If the granulosa layer of the follicle lacks the CYP11A1 coding and has no steroid activity, progesterone cannot be synthesized (14). Current research shows that water bacteria pollution can be competitively reduced by supplementation with *B. subtilis* spores via the feed and addition of probiotics in pond water (15). There is a lack of research on the molecular mechanism of LPS inhibition of progesterone synthesis. In summary, the granulosa layer secretes progesterone, which is necessary for the secretion of estrogen and testosterone by the thera cells. Therefore, it is important to study the molecular mechanism by which LPS inhibits progesterone synthesis in granulosa cells. Previous studies have shown that LPS directly affects the ovary and interferes with steroid production *in vitro* (16). In addition, it has been shown that progesterone synthesis is more susceptible to LPS in chicken pre-hierarchical follicular granulosa cells (17).

MicroRNAs (miRNAs) are a class of short-stranded non-coding RNAs with approximately 20 nucleotides in length and occupy 1%−3% of the genome. It mainly affects the genetic expression of genes by binding to mRNA targets, controlling mRNA stability, inhibiting mRNA transcription, or degrading mRNA targets (18, 19). In recent decades, new perspectives have been brought to the study of the reproductive system by the rapid growth of post-transcriptional mechanistic studies, particularly of miRNAs. Related studies have been conducted to explore miRNAs with important roles in follicle development from the expression profiles of identified miRNA populations (20). The miRNAs expressed in the ovary are involved in the regulation of granulosa cell progesterone synthesis (21). Sirotkin et al. reported that of 80 human pre-miRNAs studied, 36 were engaged in inhibiting granulosa cell progesterone synthesis, and 10 miR-10a-5p and miR-103a-3p affected oocyte development by targeting BDNF (22). Several studies have identified miR-10a-5p as a key regulatory miRNA for ovarian function. miR-10a-5p was found to inhibit progesterone production by down-regulating CDK2 in chicken granulosa cells (23). Meanwhile, miR-10a-5p inhibited steroidogenesis by targeting CREB1 in porcine granulosa cells (24). It also plays a role in ovarian cancer (25). Similarly, there is still plenty of room for research on functional miRNAs for ovarian disorders in avian species. Studies on miRNAs have focused on mammals, but in avian species, miRNA regulation of progesterone has been demonstrated.

In summary, LPS causes a decrease in animal performance and harms animal husbandry. miR-10a-5p is a key molecule involved in ovarian development and progesterone regulation. This study aimed to determine the molecular mechanism of miR-10a-5p involved in LPS reducing progesterone synthesis in goose granulosa cells, to reveal the mechanism of the miR-10a-5p-mediated effect of LPS on goose egg production performance through *in vivo* and *in vitro* experiments, and to lay the theoretical foundation for reducing the harm of LPS on poultry breeding.

## 2 Materials and methods

### 2.1 Establishment of animal model and groups

All experimental procedures were undertaken according to the Administration of Animal Care and Use guidelines and were approved by the Animal Ethics Committee of Nanjing Agricultural University, Nanjing, Jiangsu, China.

Yangzhou goose was used as the experimental animal in this study. All the geese were raised in cages under the same environment with *ad libitum* conditions. Breeding geese of the same batch and similar weight at the peak of egg-laying were selected, the whole egg-laying process was monitored, and the laying period of the geese was precisely recorded and marked with wing markers. A pre-experiment was conducted to establish the optimal dose. LPS (from *E. coli* O55:B5) injection and slaughtering time were determined based on the protocol of previous experiments in our laboratory (7). The geese were injected with LPS (1.5 mg/kg BW) intravenously at 8 h, 32 h, and 44 h after egg laying and slaughtered at 0 h, 12 h, and 24 h of LPS Exposure, respectively, for the breeder geese injected with LPS 8h after egg laying were slaughtered 8 h after egg laying as Control group. At the same time, the breeder geese injected with LPS 32 and 44 h after egg laying were slaughtered 8 h after the next egg laying. The experimental animals were divided into three groups, including the group that had LPS treatment for 0 h (L0), 12 h (L12), and 24 h (L24).

### 2.2 Follicular granular layers collection and RNA extraction

Total RNA was extracted from the goose follicular granular layers using TRIzol^®^ reagents following the manufacturer's manual (Invitrogen, Carlsbad, CA). Degradation and contamination of the total RNA were detected on 1% agarose gels. The purity of the total RNA was assessed using a NanoPhotometer^®^ spectrophotometer (IMPLEN, CA). The integrity was estimated using an RNA Nano 6000 Assay Kit with the Agilent Bioanalyzer 2100 system (Agilent Technologies, Santa Clara, CA). The RNA concentration was checked with a Qubit^®^ RNA Assay Kit in a Qubit^®^ 2.0 fluorometer (Life Technologies, CA). The 28S/18S ratio of the qualified RNA ranged from 1.8 to 2.0, and the RNA integrity values ranged from 8.0 to 10.0. RNA samples were stored at−80°C for further analysis. The follicles of 18 individual geese (*n* = 6) were collected. After slaughtering the breeding goose, the abdominal cavity was dissected, and all the follicles were removed. The removed follicles were placed in trays with saline, the different grades of follicles were separated and placed in Petri dishes with saline, and the outer follicular membrane, connective tissue, and vascular network were stripped clean. The granular layer was frozen in liquid nitrogen and stored at −80°C before RNA extraction.

### 2.3 Cell transfection and treatment

Primary goose granulosa cells (GCs) were obtained from F1–F3 hierarchical follicles of Yangzhou geese at peak laying period. Cells were cultured in M199 medium (Gibco, USA) with 10% FBS (Gibco, USA) and 1% penicillin-streptomycin (Solarbio, China) for 24 h. The transfection experiment was carried out according to the concentration recommended by the manufacturer when the density of follicular granulosa cells in a 6-well plate was ≥80%. The new complete medium was changed after transfection for 36 h. To be specific, cells were transfected with 5 μL of 20 μM miR-10a-5p mimics, 5 μL of 20 μM miR-10a-5p inhibitor, and 3 μL Lipofectamine 3,000 in 1 mL Opti-MEM. Simultaneously, mock negative control (NC) transfected cells were used as a control. The sequences were F: 5′–UACCCUGUAGAUCCGAAUUUGUG–3′; R: 5′–CAAAUUCGGAUCUACAGGGUAUU–3′, 5′–CACAAAUUCGGAUCUACAGGGUA–3′, and 5′–CAGUACUUUUGUGUAGUACAA–3′, respectively. After 48 h of transfection, the original medium was discarded and replaced with a medium containing 1 μg/mL LPS for 24 h of incubation.

### 2.4 Reverse transcription quantitative real-time PCR

First-strand cDNA was synthesized using the PrimeScript™ RT Reagent Kit with gDNA Eraser (TaKaRa, Dalian, China). The RT-qPCRs were performed on a LightCycler^®^ 96 Real-Time PCR system (Roche Applied Science) in a 20 μL reaction volume containing 2 μL cDNA, 10 μL 2 × SYBR^®^Premix Ex Taq™II (TliRNaseH Plus) (TaKaRa), 0.4 μL 50 × ROX Reference Dye1, 0.3 μL each of forward and reverse primers (10 μM) and 8μL deionized water. The β-actin gene was used as a reference gene, and all RT-qPCR gene-specific primers were designed using Oligo 6.0 software. β-actin and U6 were used as reference genes, and the primer sequences are shown in Table 1. The qPCR amplification procedure was as follows: 95°C for 30 s, 40 cycles of 95°C for 10 s, and 60°C for 34 s. All reactions were performed with three replicates, and the relative gene expression levels were analyzed using the comparative CT method (also referred to as the 2^−ΔΔCT^ method).

**Table 1:** Primers used for quantitative real-time PCR.

**Gene**	**Primer Sequences (5^′^to 3^′^)**	**Annealing**
*β-actin*	F:TTACAGCAGCTCCTTGAGCC R:CTGTCGAAGGGCGAAGAAGT	60°C
*CYP11A1*	F:AACGTGCACAACATCATGGC R:ATCATCCCCTCCGACCTGAA	60°C
*STAR*	F:GGAGCAGATGGGAGACTGGA R:CGCCTTCTCGTGGGTGAT	60°C
*SCAP*	F:ACTGCCTCACCGTTATTCCCAAAC R:CGTCCAGACTGTTCTTGCCATCC	60°C
*SMAD2*	F:TTGCCATTCACTCCACCAGTTGTC R:TCCTCCTGACCATTCTGCTCACC	60°C
*BDNF*	F:AACTCCCAGTGCCGAACTACCC R:ATAAACCGCCAGCCAACTCTCTTC	60°C
*PTEN*	F:GCAGAGAGGCTTGAAGGAGTGTAC R:TGGCGGTGTCGTAATGTCTTTCAG	60°C
*GATA6*	F:GCGTCAACTGCGGCTCCATC R:GGCACTCTCTTCTGAGGCTTGATG	60°C
*GATA4*	F:TTACAGCAGCTCCTTGAGCC R:CTGTCGAAGGGCGAAGAAGT	60°C
*U6*	F:ACAAGGAGTTGAGCAAGTAGTGC R:GGTICIGCICGAATGTTCCCC	60°C
*miR-10a-5p*	F:CGCTACCCTGTAGATCCGAATTTGTG	60°C

### 2.5 ELISA analysis

We determined the concentrations of the reproductive hormone progesterone in the serum and the level of progesterone in the cell supernatant using progesterone enzyme-linked immunosorbent assay (ELISA) kits following the manufacturer's instructions. The values are presented as mean ± SEM.

### 2.6 Statistical analysis

The SPSS 26.0 software was used to perform one-way ANOVA followed by Tukey's multiple comparison tests, and the Student's *T*-test was used to determine if there were differences between two groups. All experiments were performed at least three times. Data are presented as means ± standard error of the mean (SEM). There were considered to be mean differences when there were *P*-values of < 0.05 and 0.01.

## 3 Results

### 3.1 LPS increases miR-10a-5p expression and inhibits progesterone synthesis

**Figure 1:**
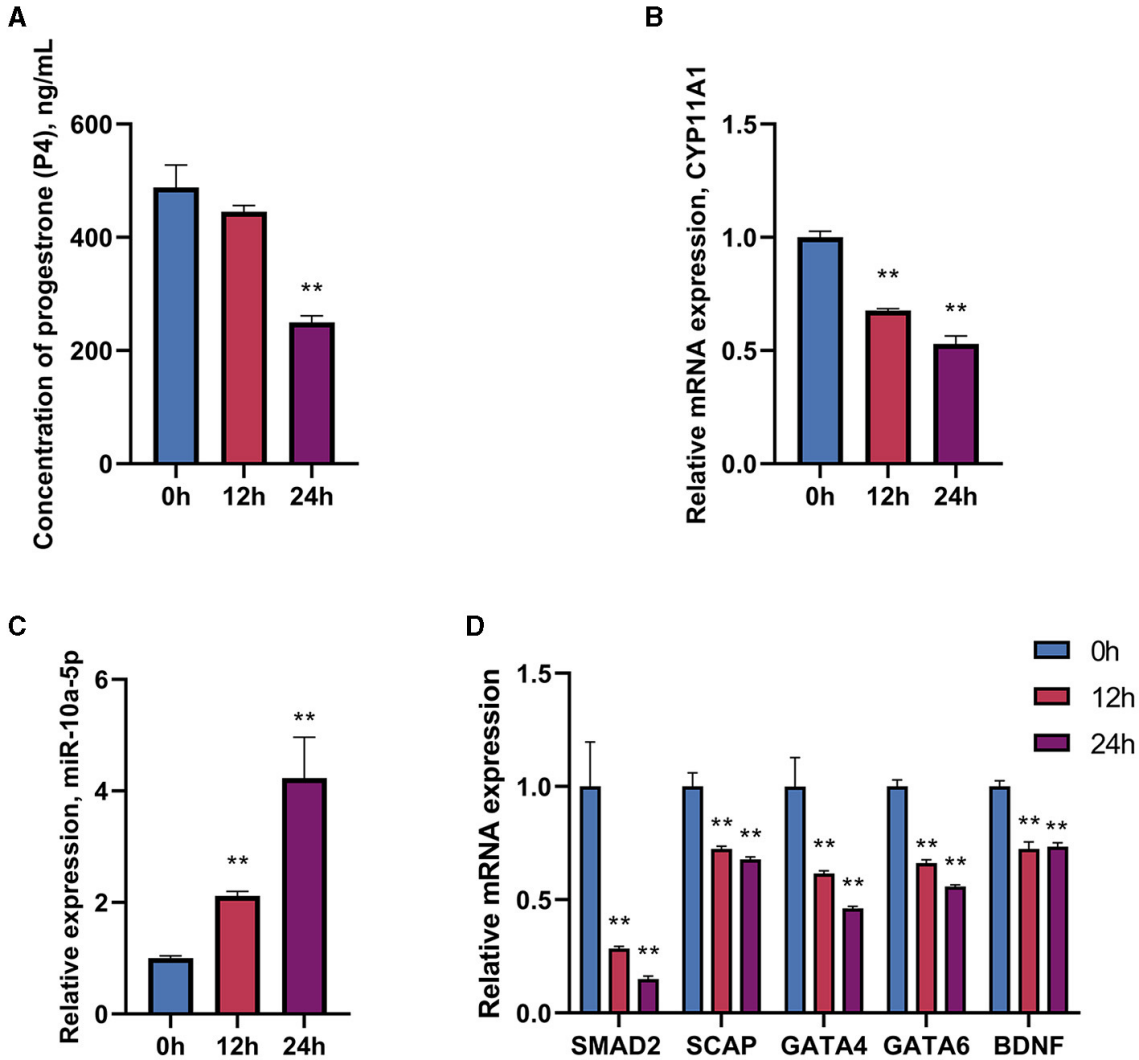
Effect of LPS on miR-10a-5p and progesterone synthesis. Geese were injected with LPS (1.5 mg/kg) for 0, 24, and 36 h, and plasma progesterone level was examined by Elisa **(A)**. CYP11A1 expression level **(B)**, miR-10a-5p expression level **(C)**, and progesterone synthesis relative genes and transcription factors expression level **(D)** were examined by RT-qPCR. Data are presented as mean ± SEM, *n* = 6. ^**^P < 0.01.

To investigate the impact of LPS on progesterone synthesis in breeding geese during the peak egg-laying period, geese were treated with LPS (1.5 mg/mL) for 0, 12, and 24 h. The expression of progesterone-related genes, transcription factors, and regulatory factors was detected using RT-qPCR, and the concentration of progesterone in serum was measured using ELISA. Following serum LPS treatment, there was a tendency for progesterone levels to decrease after 12 h. At 24 h, progesterone levels decreased significantly compared to the control group (Figure 1A). The expression of CYP11A1, a gene encoding the progesterone50SCC protein, was also significantly reduced (Figure 1B). Additionally, miR-10a-5p expression was affected by LPS treatment and increased with increasing treatment time (Figure 1C). The expression of transcription factors SMAD2, SCAP, PTEN, GATA6, and BDNF related to progesterone synthesis in GCs was significantly reduced by LPS treatment (Figure 1D). Additionally, miR-10a-5p and genes related to progesterone secretion showed a sustained decrease in expression levels after LPS stimulation in a time-dependent manner. The data indicate that in the *in vivo* experiments, LPS stimulation at 12 h acts as a suppressor of expression for progesterone-related genes. LPS treatment for 24 h inhibited progesterone levels.

### 3.2 Effect of different doses of LPS on progesterone synthesis in goose GCs

**Figure 2:**
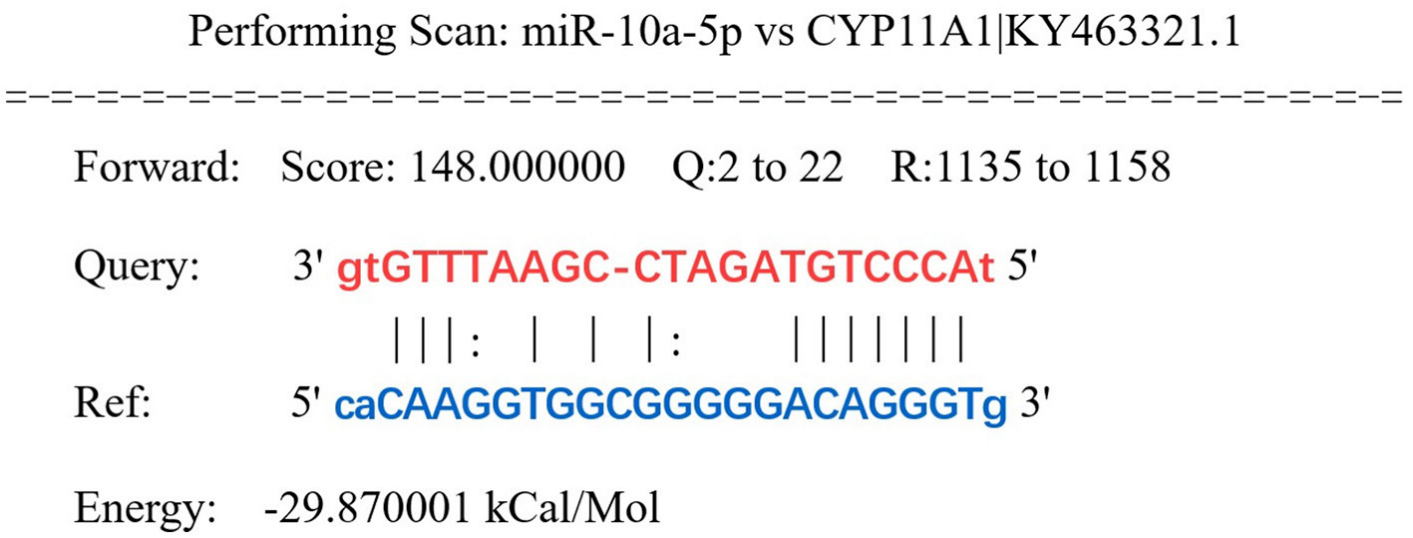
CYP11A1 was predicted as a target gene of miR-10a-5p. Highly conserved binding sites of miR-10a-5p (MIMAT0046422) and CYP11A1 were predicted by miRanda.

**Figure 3:**
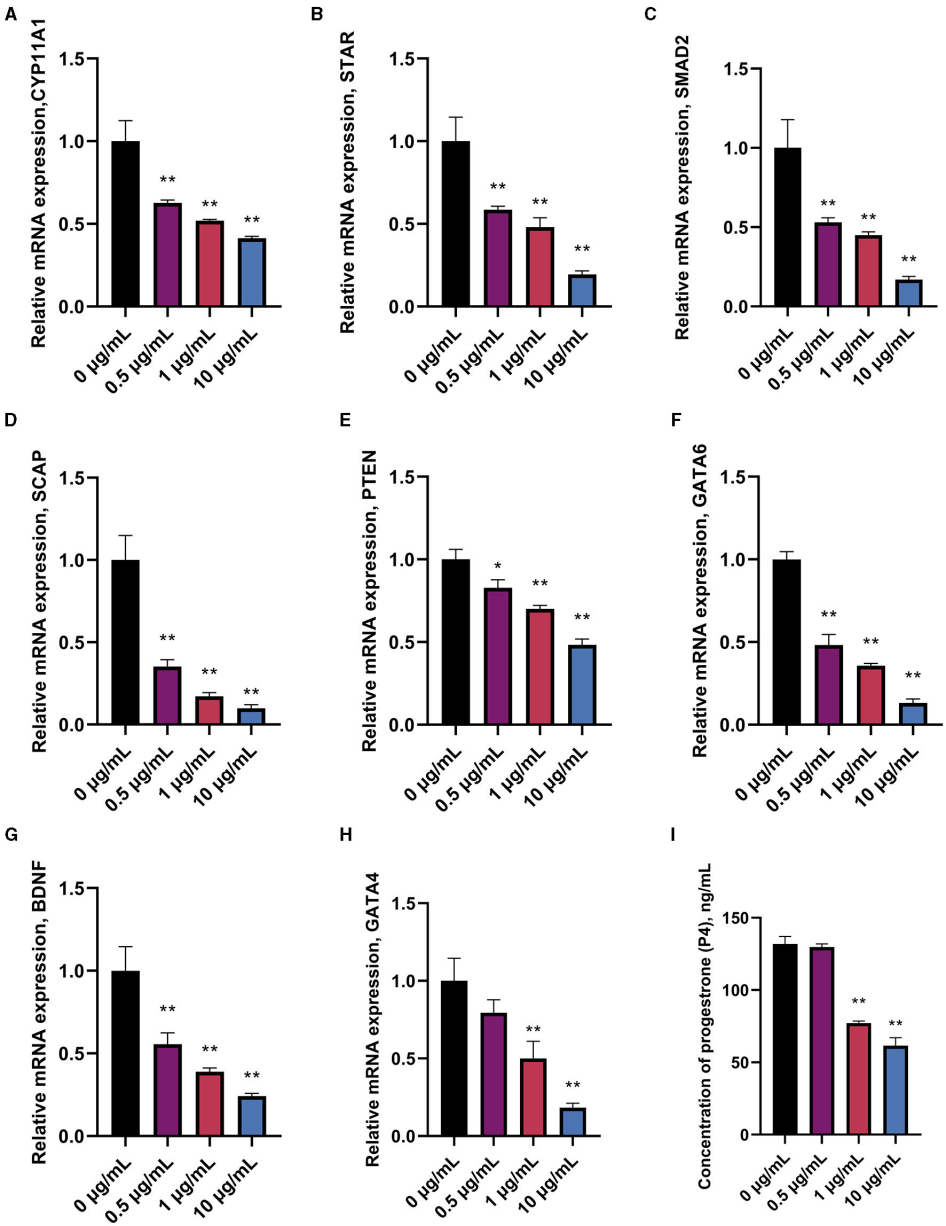
Effect of different treatment concentrations of LPS on progesterone synthesis in goose GCs. GCs were treated for 24 h with 0, 0.5, 1, and 10 μg/mL of LPS, and mRNA expression of CYP11A1 **(A)**, STAR **(B)**, SMAD2 **(C)**, SCAP **(D)**, PTEN **(E)**, GATA6 **(F)**, BDNF **(G)**, and GATA4 **(H)** were examined by RT-qPCR. **(I)** Progesterone level was tested by ELISA. Data are presented as mean ± SEM, *n* = 9. ^*^P < 0.05. ^**^P < 0.01.

Prediction of miRNAs with targeting relationship to CYP11A1 using miRanda 3.3a bioinformatics revealed that miR-10a-5p has a conserved binding site to CYP11A1 (Figure 2). To test the potential function of miR-10a-5p in regulating progesterone secretion by GCs, we established primary goose granulosa cell cultures and examined the effect of miR-10a-5p on an *in vitro* model of LPS-induced injury. GCs were exposed to different doses of LPS (0.2, 0.5, 1, 2, and 5 μg/mL); ELISA was used to determine the concentration of progesterone in the culture broth. LPS significantly decreased relevant genes in GCs at different concentrations (Figures 3A–H). We observed that different genes were sensitive to LPS to various degrees. CYP11A1, SMAD2, SCAP, STAR, PTEN, GATA4, and BDNF were significantly decreased at a concentration of 0.5 μg/mL. Showed a significant decrease at a concentration of 1 μg/mL, whereas GATA4 was the least sensitive to the concentration of LPS and showed a significant reduction at 1 μg/mL LPS treatment. Similarly, we observed a dose-dependent down-regulation of progesterone content in the culture medium (Figure 3I). The above data suggest that LPS induction inhibits progesterone production in GCs *in vitro*. Since the progesterone concentration showed a significant decrease at 1 μg/mL LPS treatment, this concentration was selected as the treatment concentration for subsequent experiments.

### 3.3 Effect of LPS treatment for 36 h on progesterone synthesis in goose GCs

**Figure 4:**
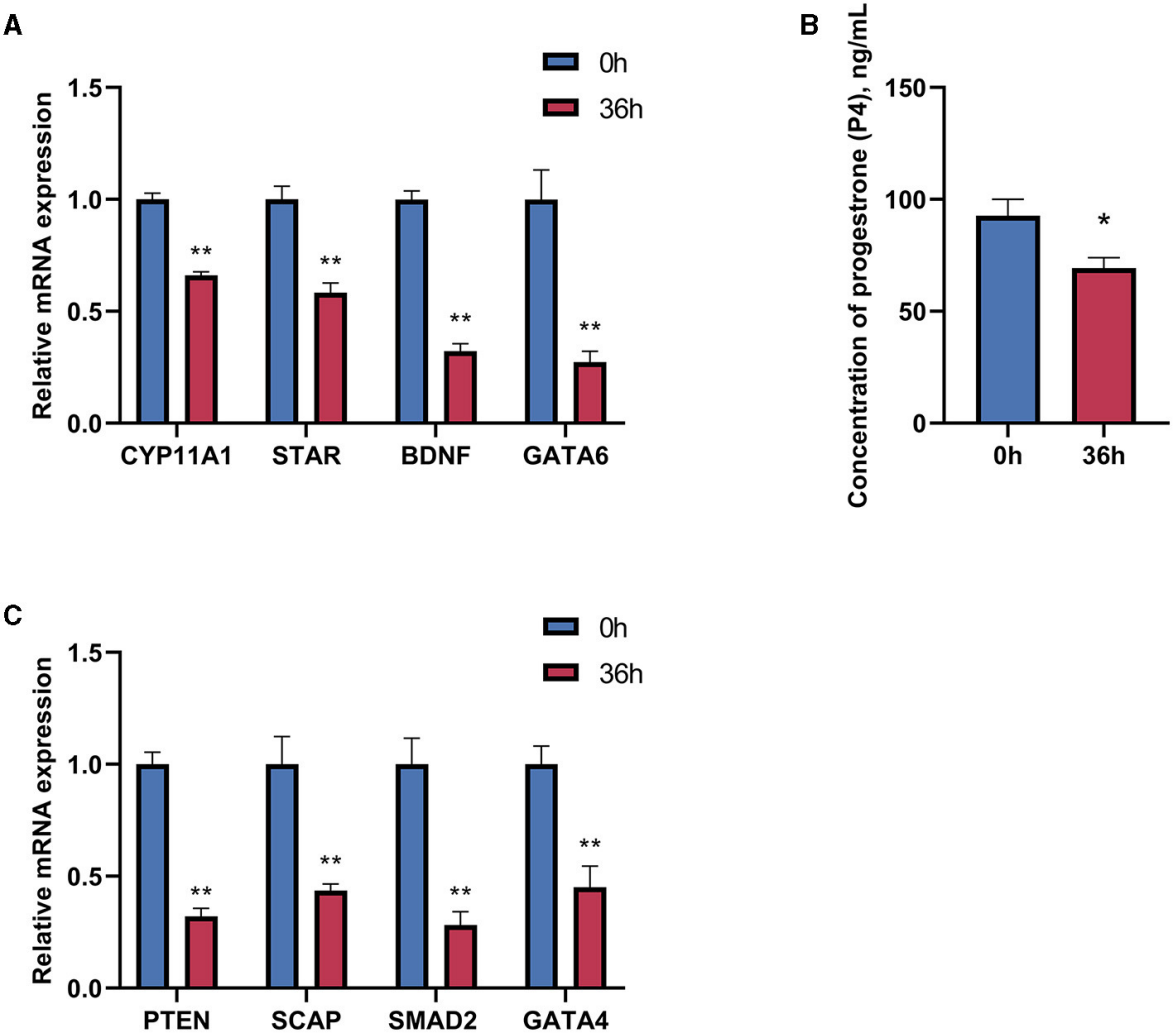
Effect of LPS treatment for 36 h on progesterone synthesis in goose hierarchical follicular GCs. **(A)** After 36 h of 1 μg/mL LPS stimulation, the expression levels of CYP11A1, STAR, BDNF, and GATA6 were tested. **(B)** Progesterone level was tested by Elisa. **(C)** The expression levels of PTEN, SCAP, SMAD2, and GATA4 were tested. Data are presented as mean ± SEM, *n* = 9. ^*^P < 0.05, ^**^P < 0.01.

To evaluate the effects of time and dose in the *in vitro* experiments, the treatment time of GCs was extended to 36 h based on the provided data. The expression of genes related to progesterone synthesis was measured under the stimulation of 1 μg/mL LPS. The results showed significant decreases in the expression of progesterone-related genes, regulatory and transcription factors, and the target gene *CYP11A1* (Figures 4A, B). Furthermore, there is a significant decrease in the concentration of progesterone in the supernatant of the cell culture fluid with prolonged treatment time of LPS, indicating a substantial impact on the inhibitory effect of LPS (Figure 4C).

### 3.4 miR-10a-5p inhibits progesterone production

**Figure 5:**
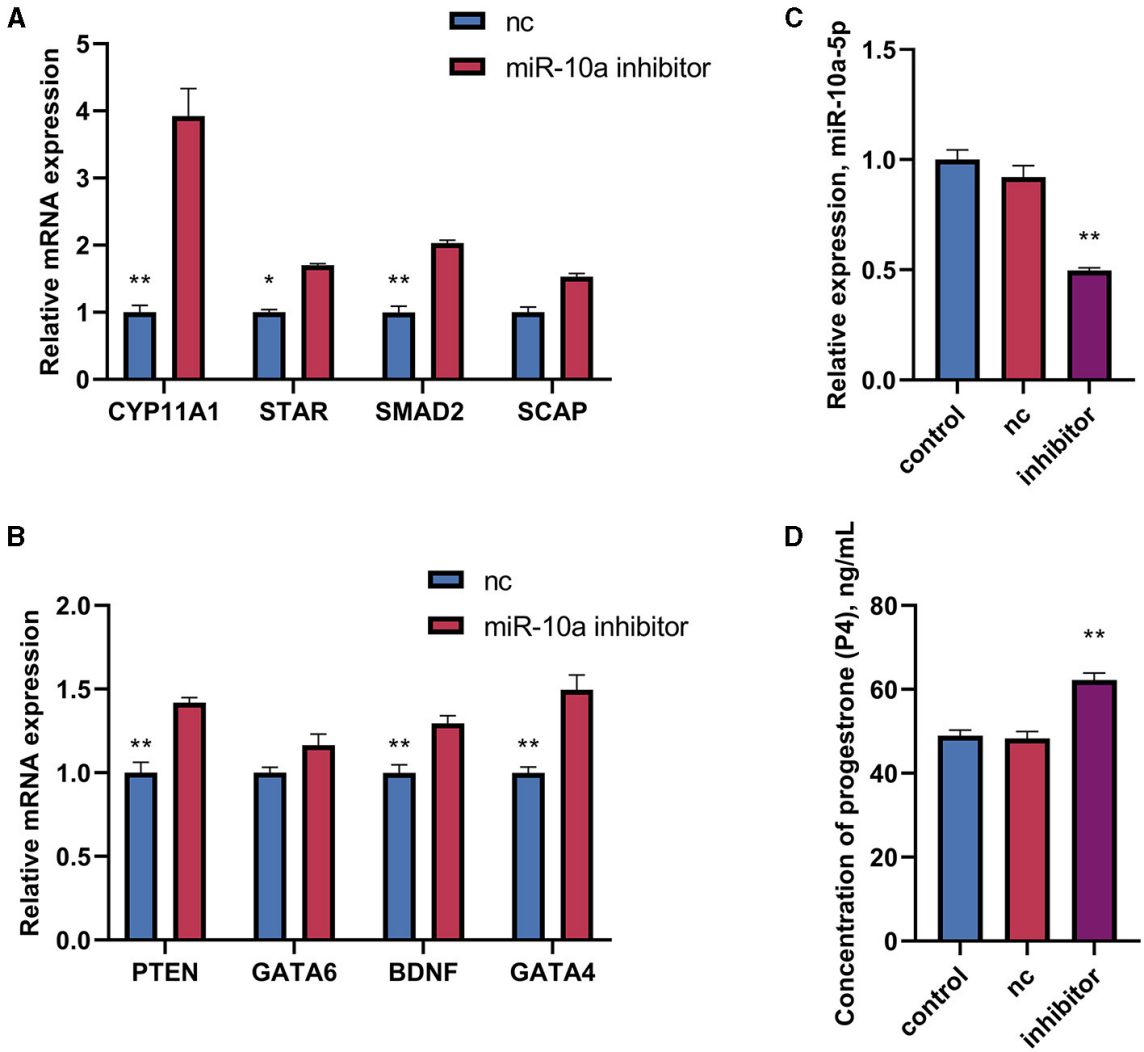
The effect of inhibiting miR-10a-5p and LPS on the expression of progesterone synthesis-related genes and progesterone levels in goose GCs. MiR-10a-5p mimics were transfected into GCs, the expression of **(A)** CYP11A1, STAR, BDNF, and GATA6 levels, and **(B)** PTEN, SCAP, SMAD2, and GATA4 levels were tested. **(C)** The transfection efficiency of miR-10a-5p inhibitor was verified by RT-qPCR. **(D)** Progesterone level was tested. GCs transfected with negative sequences were used as control group (nc). Data are presented as mean ± SEM, *n* = 9. ^*^P < 0.05. ^**^P < 0.01.

**Figure 6:**
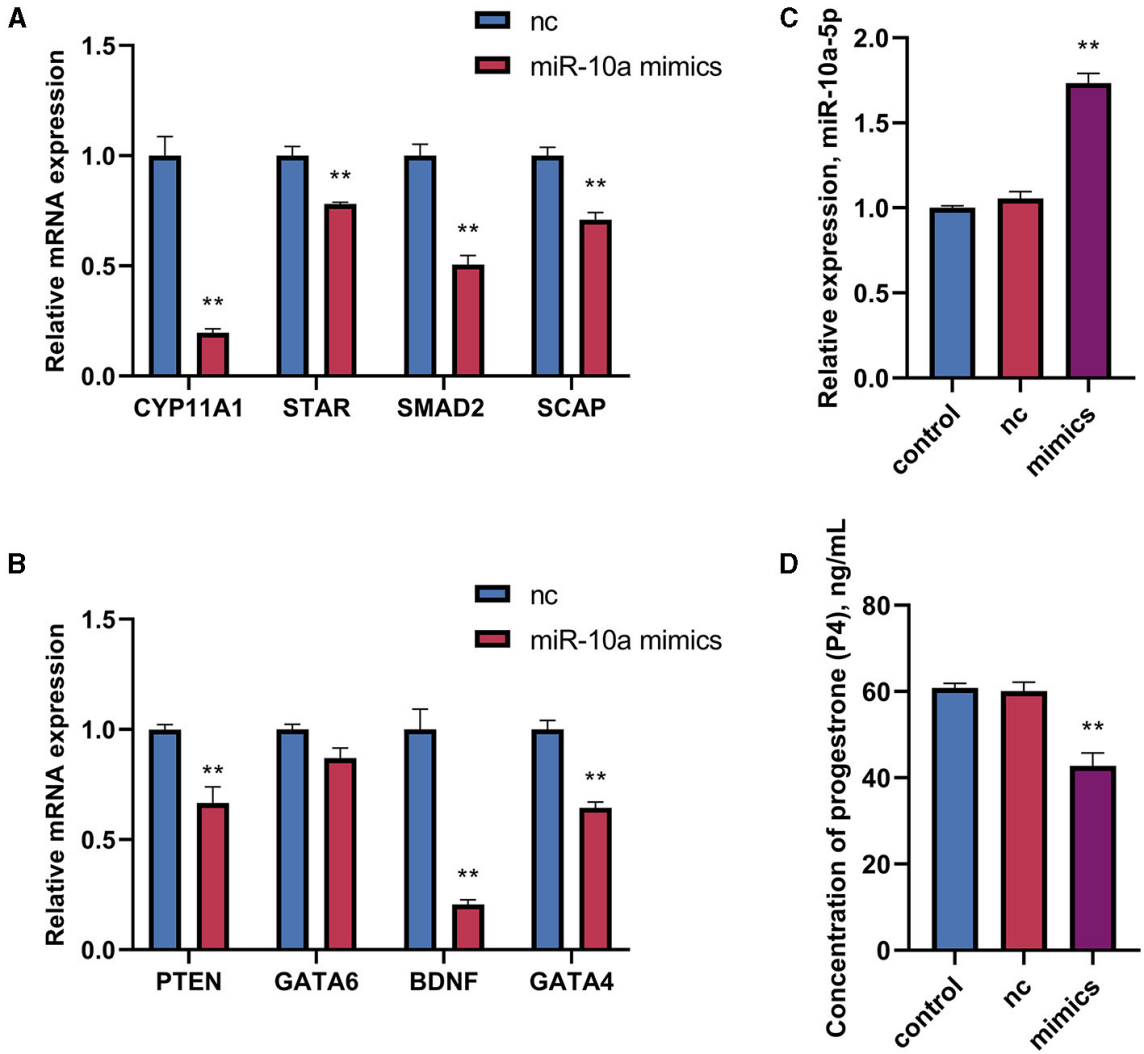
The effect of overexpression of miR-10a-5p on the expression of progesterone synthesis-related genes and progesterone level in goose GCs. MiR-10a-5p mimics were transfected into GCs, the expression of **(A)** CYP11A1, STAR, BDNF and GATA6 levels were tested. **(B)** The expression PTEN, SCAP, SMAD2, and GATA4 levels were tested. **(C)** The transfection efficiency of miR-10a-5p mimics was verified by RT-qPCR. **(D)** Progesterone levels was tested. GCs transfected with negative sequences were used as control group (nc). Data are presented as mean ± SEM, *n* = 9. ^**^P < 0.01.

After confirming a potential association between miR-10a-5P and CYP11A1, we further investigated the role of miR-10a-5P in regulating progesterone. We achieved low expression or overexpression of miR-10a-5P by transfecting a miR-10a-5P inhibitor or miR-10a-5P mimic vector, verified by RT-qPCR assay (Figures 5A–C). No significant difference between the blank and negative control was observed. After treating goose GCs with miR-10a-5p inhibitor or miR-10a-5p mimics for 48 h, the expression of related genes changed. Inhibition of miR-10a-5p for 48 h resulted in a significant up-regulation of CYP11A1 expression compared to the blank group. Additionally, the expression of the progesterone-related transcription factor and the progesterone synthesis gene STAR was significantly elevated compared to the blank and NC groups. There was a significant increase in the concentration of progesterone in the cell culture medium, which was negatively correlated with the miR-10a-5p inhibitor treatment (Figure 5D). The expression of the target gene CYP11A1 was significantly reduced (Figure 6A), along with the progesterone synthesis-related genes (Figure 6B). After transfection with miR-10a-5p mimics, intracellular miR-10a-5p expression was reduced, indicating effective transfection (Figure 6C). Progesterone concentration also decreased (Figure 6D).

### 3.5 miR-10a-5p is involved in LPS-induced progesterone synthesis decrease in goose GCs

**Figure 7:**
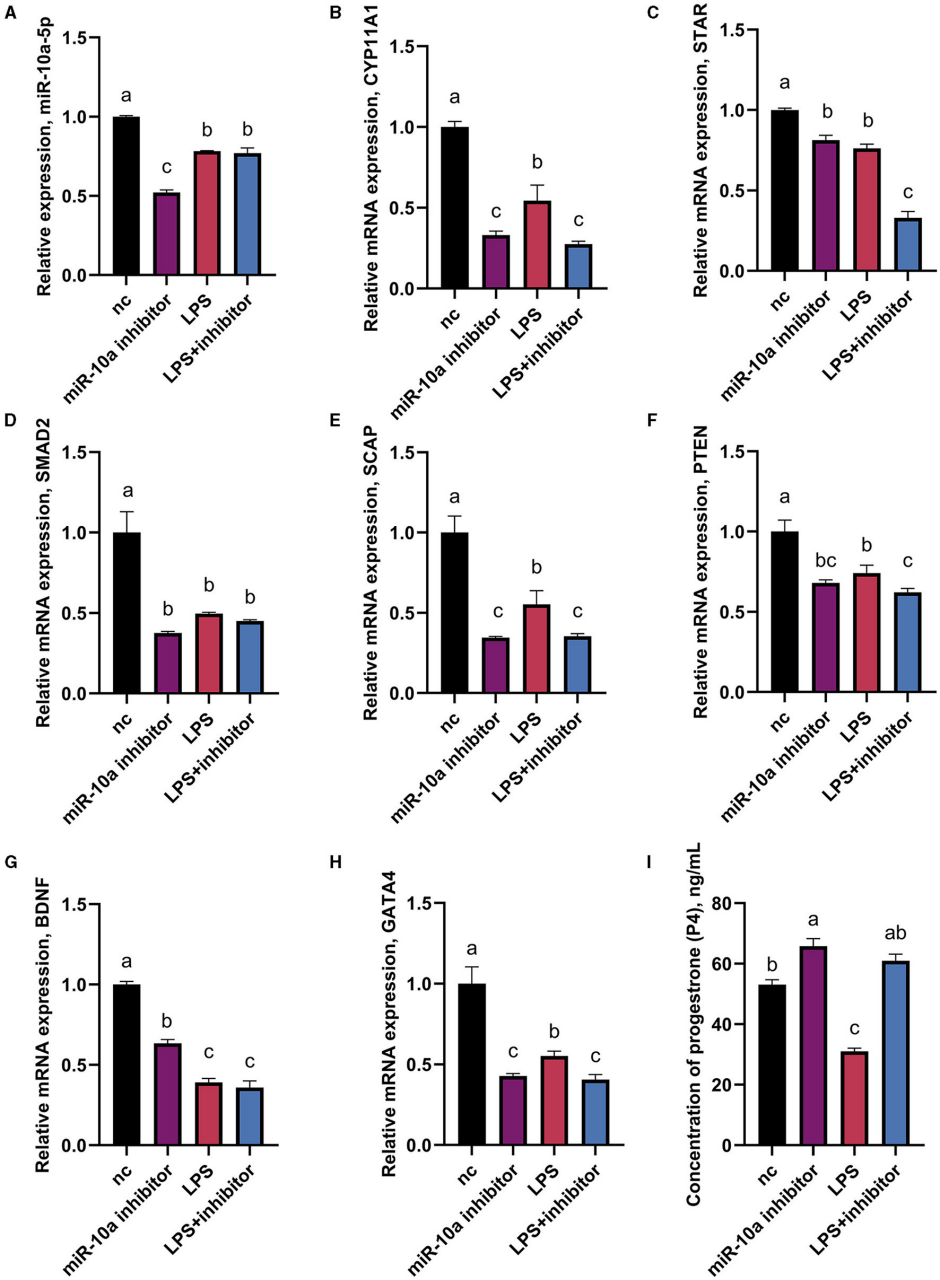
The effect of inhibiting miR-10a-5p on the expression of progesterone synthesis-related genes and progesterone level in LPS-treated goose GCs. After 36 hours of 1ug/ml LPS stimulation and miR-10a-5p inhibitor were transfected into GCs, the expression levels of miR-10a-5P **(A)**, CYP11A1 **(B)**, STAR **(C)**, SMAD2 **(D)**, SCAP **(E)**, PTEN **(F)**, BDNF **(G)**, GATA4 **(H)** were examined by RT-qPCR. **(I)** Progesterone level was tested by Elisa. GCs transfected with negative sequences were used as control group (nc). Data are presented as mean ± SEM, *n* = 9. Values with different letters are significantly different (*p* < 0.05).

**Figure 8:**
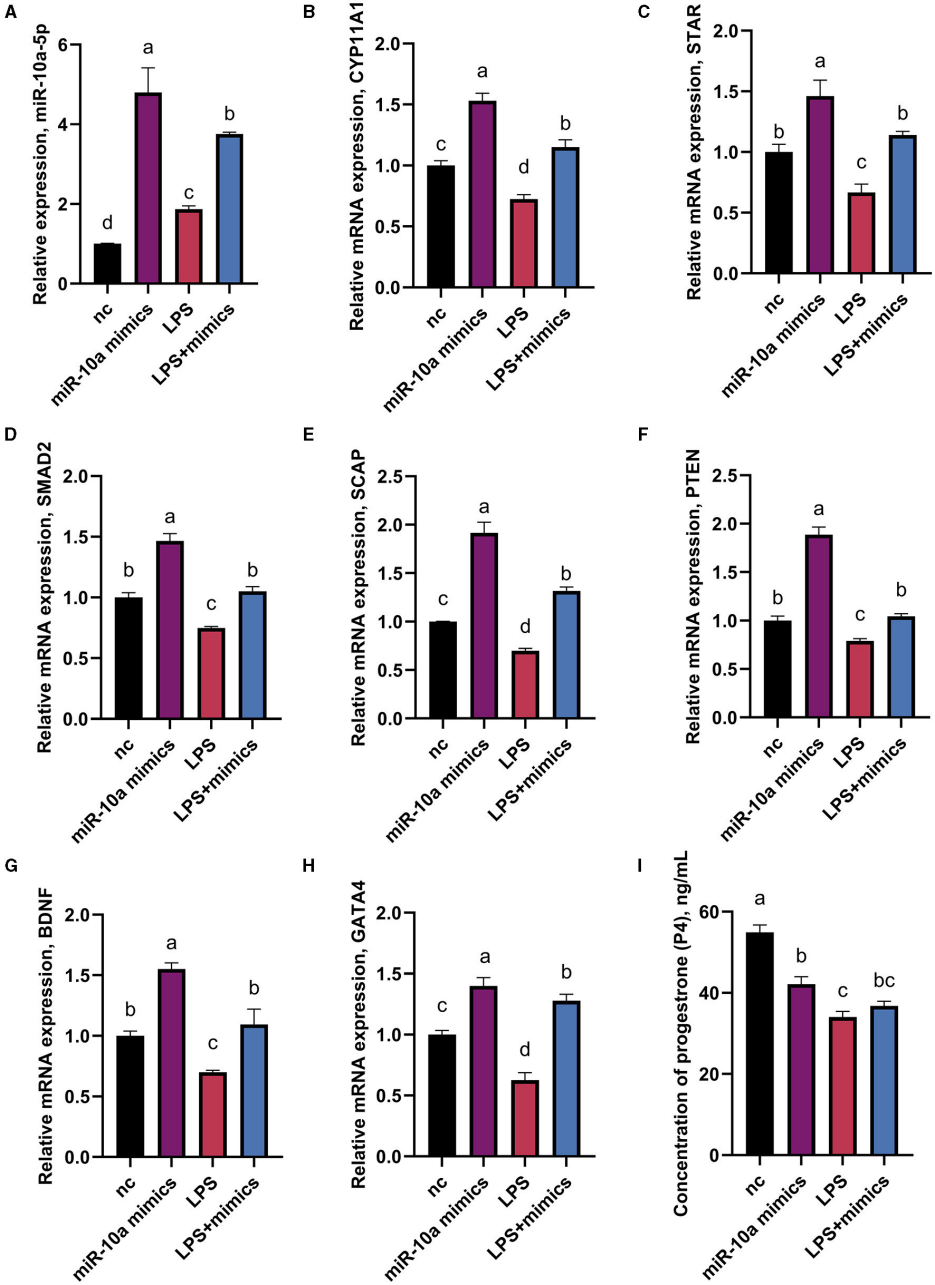
The effect of overexpression of miR-10a-5p on the expression of progesterone synthesis-related genes and progesterone level in LPS-treated goose GCs. After 36 hours of 1μg/mL LPS stimulation and miR-10a-5p mimics were transfected into GCs for 48h, the expression levels of miR-10a-5P **(A)**, CYP11A1 **(B)**, STAR **(C)**, SMAD2 **(D)**, SCAP **(E)**, PTEN **(F)**, BDNF **(G)**, GATA4 **(H)** were examined by RT-qPCR. **(I)** Progesterone level was tested by Elisa. GCs transfected with negative sequences were used as control group (nc). Data are presented as mean ± SEM, *n* = 9. Values with different letters are significantly different (*p* < 0.05).

As mentioned above, CYP11A1 is the gene encoding P450SCC, a key factor in producing progesterone and sex hormones, including estrogen and testosterone. RT-qPCR and ELISA were used to determine gene expression and progesterone hormone levels. Since GATA6 was not affected by the expression of miR-10a-5P in the previous experiment, the expression of other seven progesterone-related genes was verified. GCs were treated with 1 μg/mL LPS for 36 h, and were transfected with either a miR-10a-5p inhibitor or a miR-10a-5p mimic for 48 h. After miR-10a-5p inhibitor were transfected into LPS-induced GCs, the expression level of miR-10a-5p decreased (Figure 7A). The miR-10a-5p inhibitor significantly increased the expression level of the cells, while LPS stimulation decreased the expression level (Figures 7B–H). After 36 h of 1 ug/ml LPS stimulation, the inhibitor restored the cell expression levels to normal and decreased LPS-induced expression changes in CYP11A1, SMAD2, SCAP, PTEN, GATA6, BDNF, and GATA4. Meanwhile, progesterone levels decreased due to LPS stimulation, and low expression of miR-10a-5p restored this trend (Figure 7I). On the other hand, Expression levels of miR-10a-5p increased after transfection of the miR-10a-5p mimics into LPS-induced GCs (Figure 8A). miR-10a-5p mimics significantly reduced the expression level of cells upon LPS stimulation (Figures 8B–H). LPS stimulation further enhanced the inhibitory effect of miR-10a-5p mimics to downregulate CYP11A1, STAR, SCAP, PTEN expression, and progesterone level inhibition expression (Figure 8I).

## 4 Discussion

This study investigated the mechanism of mir-10a-mediated LPS inhibition of progesterone synthesis in goose GCs by verifying the expression levels of progesterone synthesis-related genes and transcription factors. Yangzhou geese at peak laying period were selected as experimental animals in this study to investigate the mechanism of miR-10a-5p mediated LPS inhibition of progesterone synthesis in goose GCs by verifying the expression levels of genes and transcription factors related to progesterone synthesis. Geese are large waterfowls who prefer mating on water, laying a few eggs only in winter and spring, and having an annual egg production of about 70 eggs. To fill the gap in summer breeding goose production, the out-of-season egg-laying technique has been developed by our team, which allows breeding geese to lay eggs in the hot summer and increase the hatching rate through artificial incubation (26). It is a form of intensive poultry farming that maintains the right temperature, humidity, and photoperiod during the summer seasons. The economic benefits of the out-of-season egg-laying technique prompted farmers to increase stocking densities, leading to water contamination with many pathogens such as *E. coli and Salmonella* (27). The ovaries of geese have a specific mating behavior in pathogen-containing water and are susceptible to infection by pathogenic bacteria (28). Elevated plasma LPS concentrations and direct bacterial infection of the ovary reduce the egg-laying performance of geese. Our team's previous work has identified the expression pattern of the TLR receptor for LPS in hierarchical follicles (7). Therefore, in this paper, we investigated the molecular mechanism of non-coding RNAs involved in the regulation of P4 secretion by LPS after LPS directly acted on the follicle, characterized by progesterone secreted from the granulosa cell layer of the graded follicle, to explore the importance of the theoretical basis of the egg-laying pattern in the antipodes supplemented by the molecular mechanism of non-coding RNAs involved in the regulation of P4 secretion by LPS.

The effect of LPS on production performance has been a difficult but persistent problem in the livestock industry. One link between bacterial infection and ovarian dysfunction is the accumulation of lipopolysaccharides in the follicular fluid of animals with mastitis. Estradiol is reduced in GCs cultured in LPS (29), whereas GCs from animals with mastitis have altered gene expression and reduced estradiol in follicles (30). The innate immune system relies on pattern recognition receptors in mammalian cells to detect molecular patterns associated with microorganisms or pathogens, including LPS (31). In GCs, LPS-induced reductions in E2 and progesterone were accompanied by reduced expression of key enzymes related to progesterone syntheses, such as CYP11A1 and 3β-HSD (3, 32, 33). In addition, treatment of GCs with LPS impaired progesterone production and reduced mRNA expression of STAR expression (34). A recent study by Shimizu et al. showed that LPS reduced progesterone production during the luteinization of bovine ovarian follicular membrane cells and GCs and that LPS interfered with the pre-estrous process in cattle by blocking the pre-ovulatory estradiol LPS interferes with the pre-estrus process, stops the rise of pre-ovulatory estradiol and thus delays the surge of luteinizing hormone, which also affects the synthesis and release of progesterone (35). In addition, *Salmonella*'s inhibition of chicken granulosa cells growth and expression of the *CYP11A1* gene, the rate-limiting enzyme for progesterone synthesis, may be an important reason why *Salmonella* reduces egg production in chickens (36). This study examined plasma reproductive hormone levels and gene expression levels in follicular GCs of LPS-treated breeding geese at peak egg production. Additionally, we established a culture model of LPS-injured GCs. The expression of mir-10a-5p, CYP11A1, and STAR genes, as well as transcription factors GATA4 and GATA6, and regulators SMAD2, SCAP, PTEN, and BDNF were differentially reduced by LPS. The study results indicate that LPS has an inhibitory effect on granulosa cell progesterone production. However, the mechanism behind this effect remains unclear.

MicroRNAs (miRNAs) are a class of short-stranded non-coding RNAs of 20–22 nt in length that achieve gene silencing mainly through targeted binding to mRNAs. There is growing evidence that miRNAs are key factors in regulating post-transcriptional gene expression in the ovaries of mammals and birds (21). microRNAs' main functions in the ovary are manifested as (1) differential gene expression of microRNAs during follicular atresia (37) and (2) microRNAs are differentially expressed in small, medium, and large follicles (38). A variety of miRNAs are expressed and involved in almost all biological processes in the ovary, including folliculogenesis, closure, ovulation, and regression, which involve associated cell proliferation and differentiation, apoptosis, and autophagy. In the ovary, the regulation of steroidogenesis and hormone secretion by miRNAs directly affects the follicular atresia process. Steroid acute regulatory (STAR) proteins facilitate cholesterol transport and provide substrates for steroid hormone biosynthesis. miRNAs expressed in the ovary are involved in the regulation of granulosa cell progesterone synthesis. Domestic and foreign scholars have mainly studied regulating upstream signaling pathways of progesterone synthesis. miR-221 and miRNAs-143 (39, 40), miR-29b (41), miR-320 (42), miR-96 (43), and miR-122 (44) are involved in the regulation of progesterone synthesis through targeting FSH, oxytocin receptor, E2F1 and SF-1 transcription factors, FOXO1, and LRBP signaling pathways to regulate granulosa cell progesterone synthesis.

The study identified miR-10a-5p as a target-relative miRNA for CYP11A1 through bioinformatics prediction. It also revealed other effects of miR-10a-5p on the reproductive system in mammals and birds. Hsa-miR-10a-5p inhibits cholesterol biosynthesis in cells expressing uqcrb mutants, a steroid hormone precursor (45). It is also involved in the ceRNA machinery. miR-10a-5p downregulation promoted GCs proliferation and inhibited GCs apoptosis via the circINHA/miR-10a-5p/CTGF regulatory pathway (46). miR-10a-5p inhibits progesterone synthesis by targeting MAPRE1 to suppress CDK2 in Chicken GCs (23). It was discovered that miR-10a-5p was up-regulated by LPS stimulation. Overexpression and underexpression of miR-10a-5p negatively affected intracellular gene expression. Additionally, the inhibitory effect of miR-10a-5p on the progesterone synthesis pathway was preliminarily verified, which involves several pathway factors such as SMAD2, GATA4/6, and PTEN. Previous studies have demonstrated that the miR-10a-5p/PTEN axis reduces drug resistance in human esophageal squamous cell carcinoma cells (47). This multifaceted regulation suggests that miR-10a-5p is a key miRNA for progesterone synthesis and signals the involvement of possible upstream regulation that requires further exploration. Taken together, miR-10a-5p plays a critical role in regulating ovarian function, and there may be unknown roles yet to be explored.

Progesterone production is regulated by various factors within the organism. LPS has been shown to reduce ovarian function in geese by affecting the hypothalamic-pituitary-gonadal (HPG) axis, inhibiting xxx gene expression and hormone secretion. Additionally, pathway regulation within the ovary is equally important. This study found that different genes had varying sensitivities to LPS. The response of various genes to LPS stimulation varied depending on the treatment time and dosage. BDNF is an important member of the neurotrophic factor family, consisting mainly of retro-transposable neurons secreted by neuronal target cells, and its receptors are also expressed in the ovary (48). BDNF and its receptor genes can regulate the developmental process of follicles by regulating apoptosis and proliferation of GCs. BDNF and its receptor genes can promote follicular maturation by promoting the proliferation of GCs. The study found that BDNF levels significantly decreased only when GCs were treated with 5 ug/ml LPS. However, BDNF decreased with 1 ug/ml LPS when the treatment time was increased to 36 h. Improving the treatment time is more effective in suppressing BDNF expression than increasing the stimulation concentration. SMAD2 is a receptor-activated SMAD that plays a key role in regulating gametogenesis is played by SMAD2. Studies have shown that SMAD2 is expressed during ovarian gonadal development, consistently increases during early ovarian yolk genesis, and then decreases as the ovary matures (49). The immunoreactivity of SMAD2 protein is localized in the cytoplasm of follicle cells, oocytes, and primary growth stage oocytes. SMAD2 has also been found in the chicken ovary during the transition from embryo to post-hatch (50, 51). In addition, relevant *in vitro* assays show that FSH and human chorionic gonadotropin (hCG) promote SMAD2 expression in ovarian tissue in a time- and dose-dependent manner (49). PTEN is a lipid and protein phosphatase expressed in certain mammalian germ cells, embryos, and neonatal ovaries, where it is involved in follicle assembly and growth during the fetal period (52). GATA4 and GATA6 are expressed in embryonic development and adult ovaries and are major transcription factors during ovarian and follicular maturation stages. GATA4 and GATA6, which have been shown to play key roles in mammalian fetal and postnatal ovarian development (53), play a role in follicular assembly, granulosa cell differentiation, postnatal follicular growth, and luteinization. They have also been reported to play a role in embryonic development. They have also been reported to be differentially expressed in the ovaries of embryonic and post-hatching chicks or follicles of different sizes (54). This research found that the miR-10a-5p inhibitor helped the cells' expression levels return to normal during LPS stimulation. Additionally, the miR-10a-5p mimics significantly reduced the expression levels of genes, and LPS stimulation further enhanced the inhibitory effect of miR-10a-5p mimics on CYP11A1, STAR, SCAP, and PTEN release. These findings contribute to understanding the intracellular mechanisms by which LPS inhibits progesterone synthesis.

In summary, our findings tentatively verified that miR-10a-5p is involved in LPS affecting progesterone hormone secretion through down-regulation of CYP11A1 expression, which affects follicular function and reduces egg-laying performance in geese. This study provides a theoretical basis for reducing the harmful effects of LPS in poultry farming. However, the profound effects of miR-10a-5p on poultry ovarian function require further elucidation.

## 5 Conclusions

This study demonstrates that LPS affects serum hormone levels of progesterone and the expression of genes related to progesterone production in both *in vivo* and *in vitro* experiments. Different genes exhibit varying sensitivities to LPS. Bioinformatics prediction identified CYP11A1 as the target of miR-10a-5p. A culture model of LPS injury in primary granulosa cells was established *in vitro*. Transfection experiments were conducted to determine the relationship between over-expression and under-expression of miR-10a-5p and genes related to progesterone synthesis. The study found a strong negative correlation between the expression of miR-10a-5p and several regulators of progesterone synthesis, including the target gene *CYP11A1*, the cholesterol transport rate-limiting enzyme STAR, and other progesterone synthesis-related regulators such as BDNF, SMAD2, SCAP, PTEN, GATA4, and GATA6. These findings suggest that miR-10a-5p plays a role in regulating progesterone production in goose granulosa cells.

## Data availability statement

The original contributions presented in the study are included in the article/Supplementary material, further inquiries can be directed to the corresponding author.

## Ethics statement

The animal study was approved by Animal Ethics Committee of Nanjing Agricultural University. The study was conducted in accordance with the local legislation and institutional requirements.

## Author contributions

XG: Writing – original draft, Writing – review & editing. HA: Conceptualization, Writing – review & editing. RG: Investigation, Writing – review & editing. ZD: Methodology, Writing – review & editing. SY: Funding acquisition, Project administration, Supervision, Writing – review & editing. WW: Supervision, Writing – review & editing.
